# Analysis of monomer ratios in a dimethacrylate mixture using positron annihilation spectroscopy

**DOI:** 10.1038/s41598-025-04254-3

**Published:** 2025-07-01

**Authors:** Katarína Cifraničová, Ondrej Šauša, Oľga Rosskopfová, Michaela Sedničková, Helena Švajdlenková

**Affiliations:** 1 Department of Nuclear Chemistry, Faculty of Natural Science, Comenius University in Bratislava, Ilkovičova 6, 842 15 Bratislava, Slovak Republic; 2 Institute of Physics, Slovak Academy of Sciences, Dúbravská cesta 9, 845 11 Bratislava, Slovak Republic; 3 Polymer Institute, Slovak Academy of Sciences, Dúbravská cesta 9, 845 41 Bratislava, Slovak Republic

**Keywords:** Positron annihilation lifetime spectroscopy, Ortho-positronium, Near-infrared spectroscopy, Free volume, Dimethacrylates, DMTA, Materials science, Physics

## Abstract

Dimethacrylate based samples suitable for photopolymerization are usually used in mixtures with different ratios. In the case of the monomers D_3_MA and UDMA, the ideal monomer ratio of 1:1 was reported in the literature and designed as 2 M. Dimethacrylate-based photopolymers are highly cross-linked polymer networks, indispensable in applications requiring rapid polymerization processes, such as light-curable materials for dental applications. Different ratios of the starting monomers D_3_MA and UDMA in a mixture with the addition of a 0.1 mol% photoinitiator was used in near-UV light photopolymerization. The lifetime of the ortho-positronium was monitored by PALS and the local free volume were determined. NIR was used to follow the double bond transformation in the dimethacrylates region. Bulk density was determined using Archimedes’ law. The phase transitions and the glass transition temperatures (Tg´s) were determined using the dynamic mechanical thermal analysis (DMTA). Based on the free volume size, double bonds and density of the material, the most suitable ratio of monomers used in dentistry and other application was determined. Correlations between the local free volume, density, and chemical bonding of the studied samples with different ratios are discussed.

## Introduction

From electronics to medical applications, polymers are successfully coping with an ever-changing world. Their preparation is streamlined so that the resulting products are not expensive and meet the required properties. Dimethacrylate-based photopolymers form highly cross-linked polymer networks that are key for applications requiring rapid polymer processes, such as light-curable materials in dental applications. The polymers can be tailored to meet specific requirements depending on the design and synthesis. Photopolymerization is a light-induced process in which a photoinitiator reacts with a monomer under controlled conditions, resulting in the formation of a cross-linked structure. This technique is very promising in a wide range of applications such as protective and decorative coatings^1^, dental materials^2^ or 3D lithography^3^. Photopolymerization saves energy and time compared to the thermal method. One of the disadvantages of photopolymerization is the limitation of the light penetration depth, which depends on the wavelength and spectral distribution. A characteristic feature of highly cross-linked poly2M structures is the low monomer conversion at the gelation point (< 20%) and the sharp increase in stress due to shrinkage during polymerization^4^. The whole photopolymerization process is very complex and has been intensively investigated by various techniques (photo-DSC^5^, real-time infrared spectroscopy (RT-IR)^6,7^, combined near-infrared (NIR) spectroscopy^8^ with photorheometry providing information on double bond conversion (DBC) and shrinkage stress^9,10^ are also a promising method for tracking the progress of photopolymerization. The combination of multiple characterization methods contributes to a better understanding of the internal structure and properties of the crosslinked material^11–14^. One of the less commonly used techniques to study photopolymerization is positron annihilation lifetime spectroscopy (PALS). This method is based on the measurement of the lifetime of positrons or their bound state with an electron, called positronium (Ps), in the material under study. The lifetime at the site of annihilation of such states depends on the electron density in the material, i.e., on the presence of local free volumes or defects^15^. Positrons or Ps trapped in these regions have prolonged lifetimes that depend on the dimensions of these free volume structures^16^. A significant advance in the PALS technique has been brought a new methodology that combines two experiments^14^, the microscopic and macroscopic determination of thermal expansion of local free and specific volumes by PALS and dilatometry, respectively, with lattice-hole theory. The free volume sizes in such materials play a key role in the mechanical and physical properties^11^. Although polymers are generally non-toxic, there are concerns about monomer residues from incomplete polymerization. Dimethacrylate-based photopolymers form highly cross-linked networks, which are essential for rapid polymerization processes such as light-curable dental materials. These samples are typically used in a variety of blends, with a 1:1 monomer ratio often referred to as ideal, and the photopolymerization process has been studied and local free volume have been determined^17^. The ratio of monomers is studied to better understand how variations in composition affect the final properties of the material. In this context, understanding the relationship between the structure and dynamics of cross-linked photopolymers over a wide temperature range is key to the development of new materials. As part of the forward-looking research, the influence of temperature is for now not examined in this article, but it will be explored in the next future studies.

## Material and methods

### Sample

**Fig. 1:**
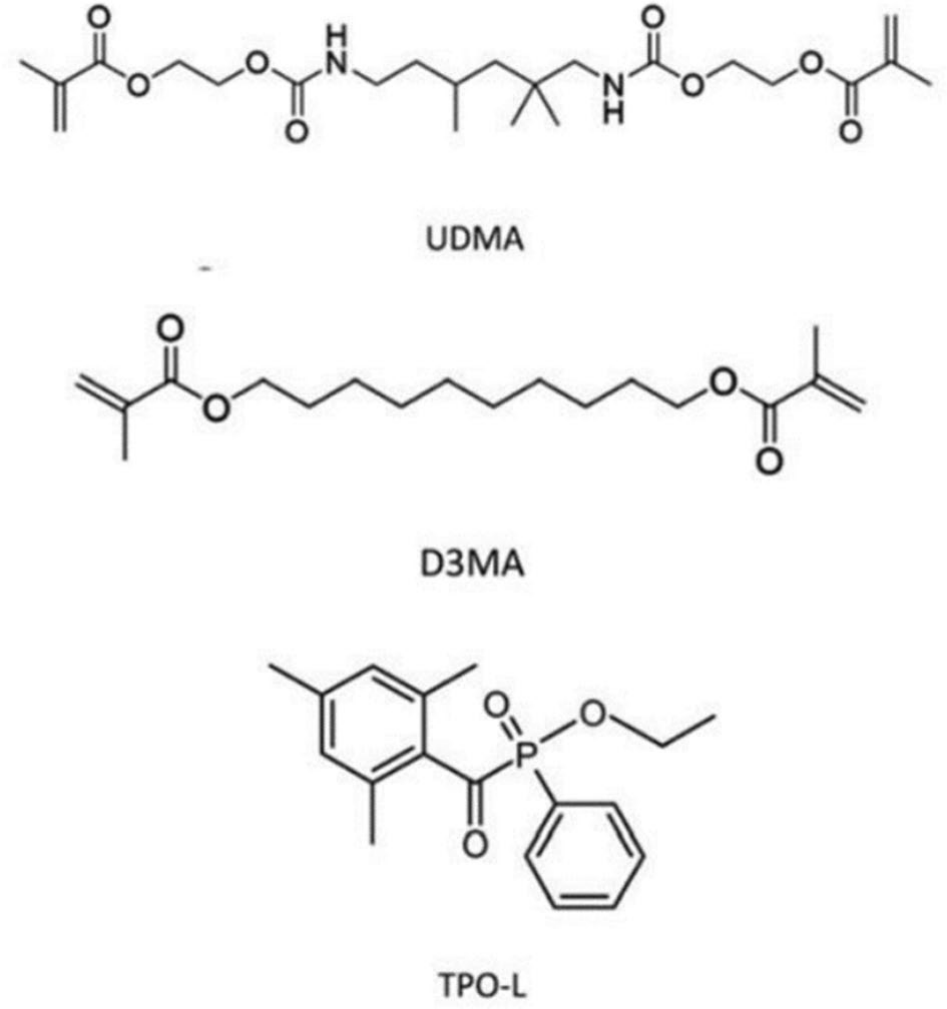
Chemical structures of UDMA and D_3_MA forming 2 M and the photoinitiator TPO-L^12^.

The mixture of dimethacrylate consists of the monomers D_3_MA (1,10-decanediol dimethacrylate, CAS: 6701–13-9, 98%) and UDMA (urethane dimethacrylate, CAS: 72869–86-4, ≥ 97%) in molar ratios of 4:1, 2:1, 1:1, 1:2, and 1:4, with the addition of 0.1 mol% of the photoinitiator TPO-L (ethyl (2,4,6-trimethylbenzoyl) phenylphosphinate), which has an absorption maximum at 380 nm. UDMA and D_3_MA monomers were purchased from Aldrich, TPO-L was purchased from TCI chemicals*, (Product Number: E0908).* The chemical structure of both monomers and photoinitiator (PI) is shown in Fig. 1. Before each experiment, mixture was homogenized for 20 min in the ultrasonic bath. All work procedures and sample handling took place in a laboratory with darkened windows. Homogenized samples were poured into a silicon ring of a thickness 2 mm with a volume of 350 μl and placed under a radioactive isotope ^22^Na as a positron source and the reference aluminium in a sandwich arrangement in PALS apparatus.

### PALS

**Fig. 2:**
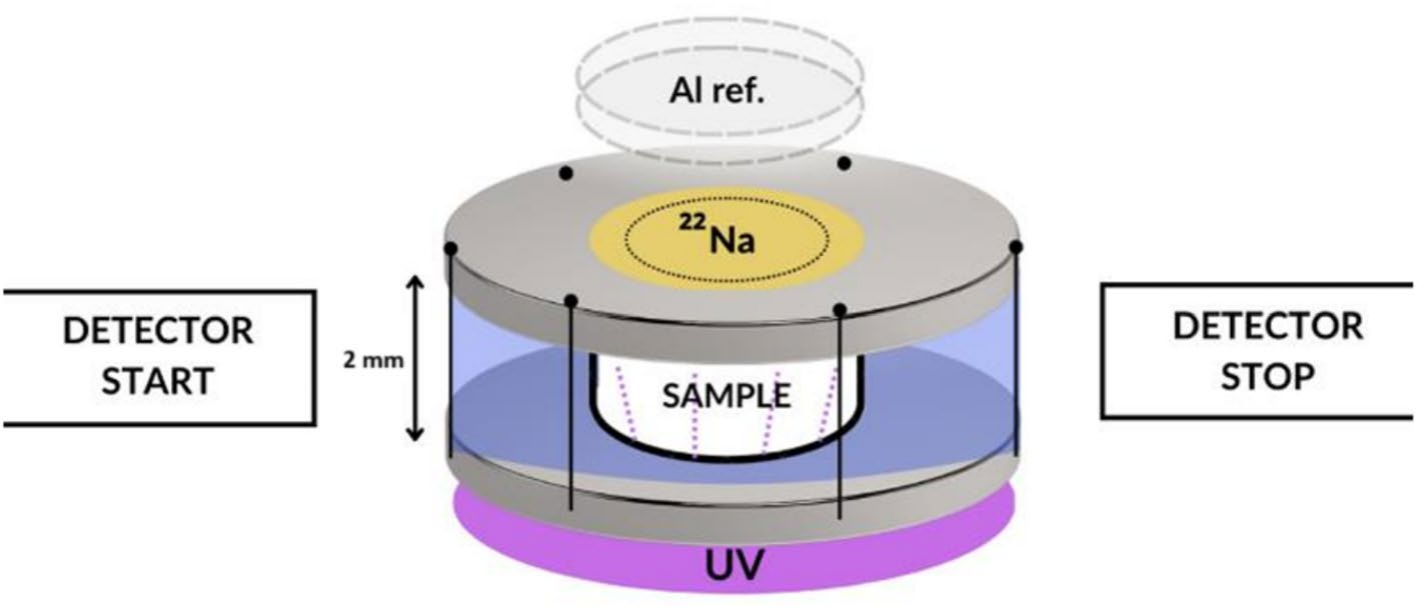
Sandwich arrangement of PALS apparatus consisting of the blue silicon ring, radioactive source ^22^Na and the reference Al^17^.

Positron annihilation lifetime spectroscopy is an experimental technique that is used in various fields of science and research to characterize the local free volume at the atomic level by measuring the lifetimes of positrons and positronium in a sample. An important process for studying the structure of a substance and its properties is the annihilation of ortho-positronium (o-Ps) through a pick-off process. A positron from an ortho-positronium atom annihilates in a solid with an electron from the surrounding environment with a suitably oriented spin. The electron–positron pair capable of annihilation is in the singlet state (S = 0) instead of the initial state (S = 1). The lifetime of the ortho-positronium is reduced from the original 142 ns to 1—4 ns depending on the structure of the material^18^. The positronium that is extinguished by the pick off process competes with the annihilation process by three annihilation quanta^18^. The probability of three-quantum annihilation is significantly smaller with respect to the pick off process. The shape and size of the free volumes in which the positronium is trapped reflect the molecular structure of the substance. In the modern world, the use of this method in the characterization of different types of materials is unique as it is the only technique that is capable of quantitatively measuring the dynamic intermolecular free volume in a substance at the molecular level^14^. Positrons are implanted into the material and the time between positron implantation and detection of annihilation radiation is measured. Positrons are usually generated using a commercially available ^22^Na radioactive positron source^19^ with an activity of 2 MBq which is sealed between two thin kapton films with the thickness of 8 μm. In sandwich configuration shown in Fig. 2, the positron source was placed between aluminium container with sample and the reference aluminium. During the conversion of the ^22^Na radioisotope atoms, there is almost simultaneous emission of a photon with an energy of 1274.5 keV. This means that if we can detect this released photon, we set the time t = 0 to measure the positron lifetime. The gamma quantum with energy 1274.5 keV, which is emitted when the positron is emitted, is used as the start signal. The stop signal is the emitted annihilation quantum with an energy of 511 keV, which is produced by the annihilation of the positron or positronium. The average lifetime of o-Ps (τo-Ps) depends on the dimension of the free-volume cavity, the size of which can be determined from a suitable Tao-Eldrup transformation model^20,21^. The PALS experiments were performed in a dark chamber at a temperature of 293.15 K. Positron annihilation lifetime spectra were obtained with a coincidence spectrometer with 25.4B12/2 M-Q-BAF-X-N detectors (Scionix, Netherland), a digital acquisition system based on a DRS4 V5 digitizer (PSI, Switzerland) and Q-PALS software^22,23^. The time resolution of spectrometer was 320 ps at FWHM (full width at the half of maximum of resolution curve). A model independent instrumental resolution function was obtained from the annihilation spectra of a defect-free Al sample with a single lifetime of 166 ps.

The light flux density at the bottom part of the silicone ring was 4.5 mWcm^-2^ for all samples. The wavelength of the light was 405 nm. The disc apparatus was placed directly on the diode light source (12 diode OSV5YL57E1A). Individual samples were measured by PALS firstly in the dark, in liquid state, before illumination. After that samples were illuminated for 1 h by a near-UV. Secondly the PALS was measured after illumination on cured samples. PALS spectra were analysed using the LT polymers^24^ program which provides data on the size of the local free volume in the sample. A typical decomposition of the lifetime spectrum is via three exponentials, which is the most common approach for polymers. The short lifetime (τ1) is related to the p-Ps lifetime, the medium lifetime (τ2) corresponds to the direct annihilation of positrons in the sample, and the longest lifetime is related to the pick-off annihilation of o-Ps (τ3, resp. τo-Ps). A correction for annihilation in the positron source was included in the LT program. The longest lifetime is used to determine the size of the local free volume^25^ and it is the subject of our analysis. Based on the longest-lived component from the annihilation of o-Ps, the average void size in a network structure is determined by the Tao-Eldrup equation^20,21^. In fact, the shape of local free volume is not spherical, but this approximation of spherical shape of local free volume is acceptable for polymers. Therefore, the mean volume of the free openings is calculated using the equation for calculating the volume of a sphere. The lifetime of ortho-positronium (τ_o-Ps_) provides insights into the intermolecular gaps within a polymer’s microstructure, which are influenced by its chemical makeup and the dynamics of its components. The size of local free volume (R_h_), where positrons are trapped, is determined by the material’s molecular structure, and then calculated local free volume (V_h_). In PALS method, the free volume is assumed to be made up of static, spherical free volume, which is an acceptable approximation for polymers.

### NIR

NIR or near-infrared spectroscopy, is a complementary technique for analysing the microstructure of polymer samples. The spectra were measured on a BW Tek detector using a BPS 2.0 light source with a spectral range of (350 to 2600) nm and a BW TEK holder, which are connected by 100 μm diameter fibre optic cables. Spectra were recorded with an integration time of 1000 ms and in the wavenumber region (4442—9431) cm^- 1^. The target of investigation was the region of double bonds (6253,10—6110,63 cm^-1^).

### Bulk density

The bulk density of the light-cured dimethacrylate samples was determined by the gravimetric method. First, the individual discs were weighed in air (m_0_) and then fixed on a copper wire and immersed in ethanol (m_1_). Their bulk density was calculated based on Eq. 1:

1 $$\rho ={\rho }_{EtOH}\left[\frac{{m}_{0}}{{m}_{0}- {m}_{1}+{m}_{wire}}\right]$$

where ρ_EtOH_ is the density of ethanol at temperature of 20—21 °C and m_wire_ is the mass of the copper wire in ethanol. This formula is based on Archimedes’ principle, which uses the difference in mass of an object in air and in a liquid to determine density. The result is a density ratio that determines the density of an unknown substance to be calculated.

### DMTA

Dynamic Mechanical Thermal Analysis (DMTA) is technic used to measure the viscoelastic properties of polymers^26^. DMTA measurements were obtained using a dynamical-mechanical analyser DMA Q800 (TA Instruments, Hüllhorst, Germany). The samples were analysed in tensile mode at a frequency of 1 Hz, a strain amplitude of 20 μm and a static force of 0.1 N in a temperature range from -50 °C to 150 °C.

## Results and discussion

**Fig. 3:**
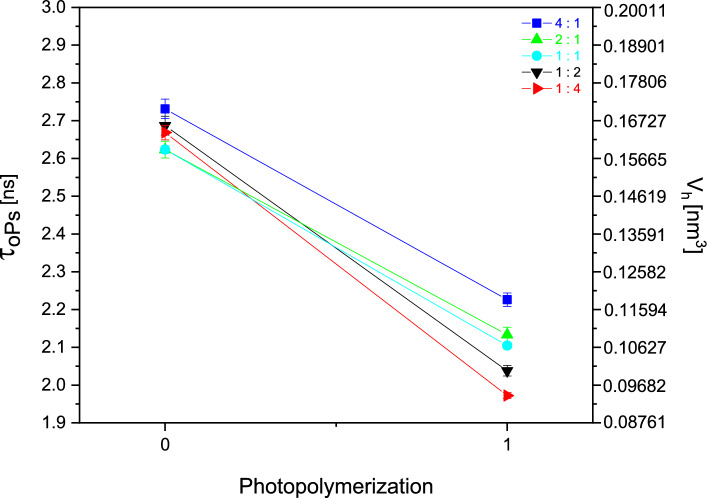
Lifetime dependence of o-Ps (τ_o-Ps_) and the associated free volume (V_h_) of the samples with 0.1 mol% PI with different ratios.

To measure the lifetime of o-Ps in local free volume using PALS, samples with different ratios of monomers (D_3_MA:UDMA) were used with a thickness of 2 mm and PI concentrations of 0.1 mol%. The samples were measured by PALS in the dark, i.e., the initial state (0), and then after illumination with near-UV light, i.e., the cured state (1), see Fig. 3.

The patented 1:1 ratio sample^27^ shows the mean o-Ps lifetime among a series of cured samples, which was already studied by our research group^28^. The local free volume of this sample is ~ 0.107 nm^3^. The other samples in the liquid state have a similar lifetime of o-Ps within the measurement error, but after curing, the 1:4 ratio sample (D_3_MA:UDMA) shows the shortest lifetime and the smallest local free volume, up to ~ 0.094 nm^3^. The initial lifetimes of the o-Ps (τ_o-Ps_ (0)) measured in the dark before illumination with UV light, are all on the same level (~ 2,7 ns) for all ratios, which is indicated in Table 1 and visible in Fig. 3.

**Table 1:** Values of measured and determined PALS (τ_o-Ps_, V_h_), and density (ρ_s_) data for samples of different monomer ratios.

Sample D_3_MA:UDMA	τ_o-Ps_ (0) [ns]	τ_o-Ps_ (1) [ns]	V_h_ (1) [nm^3^]	ρ_s_ [g∙cm^-3^]
4:1	2.731 ± 0.026	2.226 ± 0.018	0.119	1.099 ± 0.001
2:1	2.624 ± 0.022	2.133 ± 0.020	0.109	1.117 ± 0.001
1:1	2.624 ± 0.010	2.105 ± 0.009	0.107	1.133 ± 0.001
1:2	2.687 ± 0.025	2.038 ± 0.014	0.100	1.153 ± 0.001
1:4	2.669 ± 0.019	1.972 ± 0.008	0.094	1.163 ± 0.001

**Fig.4:**
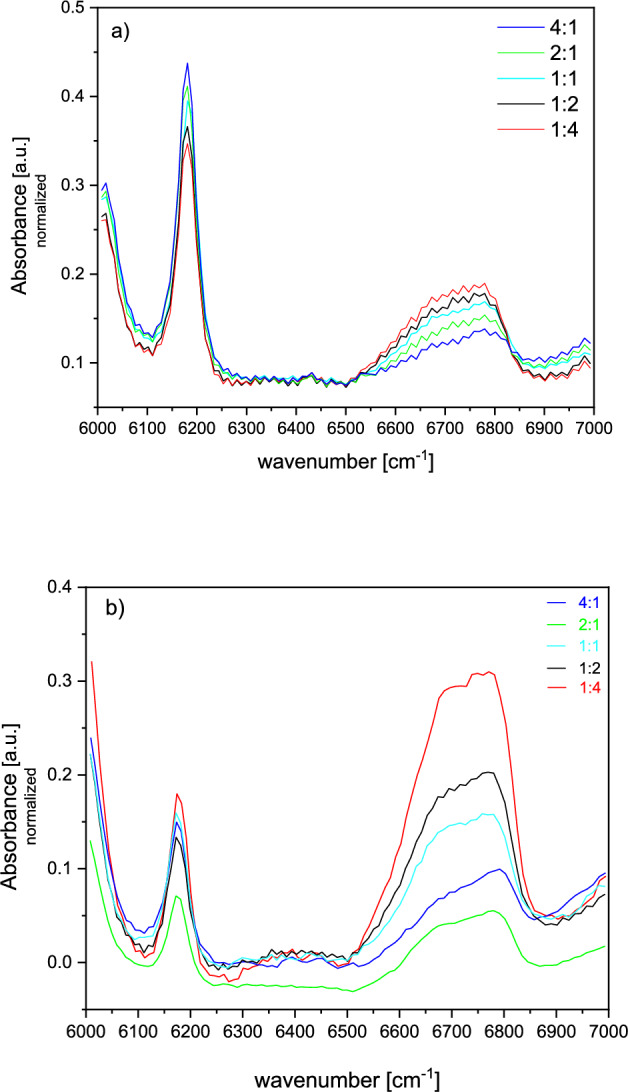
(**a, b**) Double bond region in the liquid state of the monomer blend (4a) and in the polymer after curing (4b), based on which the double bond conversion is determined (Table 2).

To confirm the dependence of the degree of double bond conversion on sample thickness and PI concentration, we subjected the samples to near-infrared spectroscopy measurements. NIR spectra of directly cured samples cured with 6.5 mW light-emitting diodes (same as for PALS) were measured at the centre of the disk (Fig. 4).

Based on the NIR spectroscopy measurement of the sample with a ratio of monomers 4:1 and density of 1.099 g/cm^3^, we can observe a bit lower double bond area of the = CH2 and higher double bond conversion (Table 2). However, the reduced bulk density suggests not so dense structure. In addition, the sample with a monomer ratio of 2:1 we can observe the lowest A_DBp_, indicating a highest efficient polymerization process supporting by high DBC (Table 2) but also lower value of bulk density. In both cases, despite the high conversion of double bonds, the larger o-Ps lifetime (τ_o-Ps_ (1), Table 1) correlates with the bulk density implying the microstructure with larger local free volumes.

**Table 2:** Analysis of NIR spectra for a series of samples, i.e., relative Area of the double bonds in after curing polymer (A_DBp_), relative Area of the double bonds in liquid state of monomer mixture (A_DBm_), double bond conversion (DBC) and bulk density of cured samples (ρ_s_).

Sample D_3_MA:UDMA	A_DBp_ [a.u.]	A_DBm_ [a.u.]	DBC [%]	ρ_s_ [g∙cm^-3^]
4:1	4.243	15.579	72.8	1.099 ± 0.001
2:1	3.867	14.768	73.8	1.117 ± 0.001
1:1	4.393	13.851	71.5	1.133 ± 0.001
1:2	4.336	13.470	67.8	1.153 ± 0.001
1:4	3.872	12.589	69.2	1.163 ± 0.001

The sample with equal ratio of monomers (1:1) showed larger A_DBp_, reduced DBC and the mean density 1.133 g/cm^3^ among this series of samples, which is agreement with middle value of τ_o-Ps_ (1), Table 1. The samples with increasing amount of UDMA monomers (1:2, 1:4) exhibited the higher double bond area and lower DBC. But higher bulk density 1.153 g/cm^3^ and 1.163 g∙cm^-3^ imply more dense structure which agree with the reduced o-Ps lifetime of cured samples (Table 1). Despite lower DBC, the structure in the samples with higher amount of UDMA is denser due to NH and OC groups, or between NH and NH groups^29,30^, which may contribute to additional stabilization of the structure.

Weighing the sample in ethanol took a short time compared to the time that would be required for the slow release of unreacted monomers from the sample volume into the ethanol. Therefore, we do not consider this phenomenon. It does not affect the values of the measured densities. At the same time, the sample shows the smallest free volume (0.094 nm^3^), indicating a highly compact structure which agrees with high bulk density, conversion of monomers and formation strong H boding interactions.

**Fig. 5:**
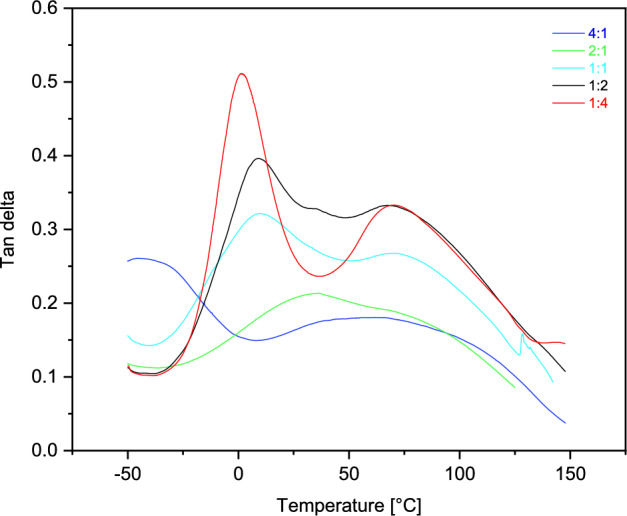
Dynamic mechanic analyzer characterization of tan δ for the samples with different monomer ratios.

A series of thermosets with different ratios of monomers measured by DMTA showed two phase transitions characterized by the glass transition temperatures (Tg), determined from the peak maxima of tanδ (Fig. 5, Table 3). Tanδ represents the ratio of loss modulus to storage modulus.

**Table 3:** Values of glass transition temperatures Tg determined by DMTA from the maxima of tanδ for a series of samples with different monomer ratios.

Sample D_3_MA:UDMA	Tg (1) [°C]	Tg (2) [°C]
4:1	55.6	112.5
2:1	33.9	89.1
1:1	9.4	72.2
1:2	9.1	66.9
1:4	1.4	70.1

In Fig. 5, the increased amount of D_3_MA monomers in a cured samples leads to the overlapped two differently high-crosslinked regions. In the case of the samples 2:1 and 4:1, two phases reflected by a broad overlapped peak were determined by deconvolution procedure. However, the samples 1:1 and the increasing amount of UDMA monomers in a crosslinked structure exhibit clear two-phase transition region. DMTA measurements showed that an increase in the amount of UDMA in the mixture decreases both Tg (1) and Tg (2), indicating higher network flexibility, while at the same time DBC decreases. On the other hand, increased UDMA content causes better packing of the polymer chains, which is well indicated by the findings of increasing bulk density, decreasing local free volume of cavities (V_h_) and strong polar interaction of NH groups for the 1:4 sample.

## Conclusions

The effect of different molar ratios of a mixture of two dimethacrylates, 1,10-decanediol dimethacrylate and urethane dimethacrylate, on the local free volume of the cured polymers determined by the PALS technique at room temperature, was studied. Measurements of the lifetimes of the o-Ps showed that increasing the UDMA monomer content decreases the lifetime and thus the decrease in local free volume. DMTA measurements showed that an increase in the amount of UDMA in the mixture decreases both Tg (1) and Tg (2), indicating higher network flexibility, while at the same time DBC decreases. NIR spectroscopy showed that an increasing amount of D_3_MA in the cured samples leads a higher double bond conversion. On the other hand, an increase of UDMA monomers in the dimethacrylate-based materials causes reductions of double bond conversion of monomers. Finally, it can be concluded that the glass transition temperatures measured by the DMTA technique indicate that a less rigid network for samples with increasing UDMA content is better spatially packed, which is well indicated by the findings of increasing bulk density and decreasing local free volume of cavities (Vh), despite lower DBC values. The strong polar interaction of NH groups also plays important role in the packaging of polymer chains. These are the key factors that determine the material properties of the cured resin.

## Supplementary Information

Supplementary Information.

## Data Availability

The data supporting this article have been included in this published article and as a part of the Supplementary Information. The datasets used and/or analysed during the current study available from the corresponding author on reasonable request.
