# Supplementary material for: Analysis of monomer ratios in a dimethacrylate mixture using positron annihilation spectroscopy
Source: Sci Rep. 2025 Jul 1;15:20494. doi: 10.1038/s41598-025-04254-3 (PMC12215763; doi:10.1038/s41598-025-04254-3)
Supplement: Supplementary file 1 — Supplementary Information. [file 41598_2025_4254_MOESM1_ESM.docx]

Analysis of monomer ratios in a dimethacrylate mixture using positron annihilation spectroscopy

Katarína Cifraničová^1*^, Ondrej Šauša^1,2^, Oľga Rosskopfová^1^, Michaela Sedničková^3^, Helena Švajdlenková^1,3*^

^1^Department of Nuclear Chemistry, Faculty of Natural Science, Comenius University in Bratislava, Ilkovičova 6, 842 15 Bratislava, Slovak Republic

^2^Institute of Physics, Slovak Academy of Sciences, Dúbravská cesta 9, 845 11 Bratislava, Slovak Republic

*^3^Polymer Institute, Slovak Academy of Sciences, Dúbravská cesta 9, 845 41 Bratislava, Slovak Republic*

^*^Correspondence to: Katarína Cifraničová (E-mail: katarina.cifranicova@uniba.sk), Helena Švajdlenková (E-mail: [helena.svajdlenkova@savba.sk](mailto:helena.svajdlenkova@savba.sk))

1. **PALS**

Representative PALS spectra for the mixture ratio 4:1 and 1:4 is displayed for the comparison.


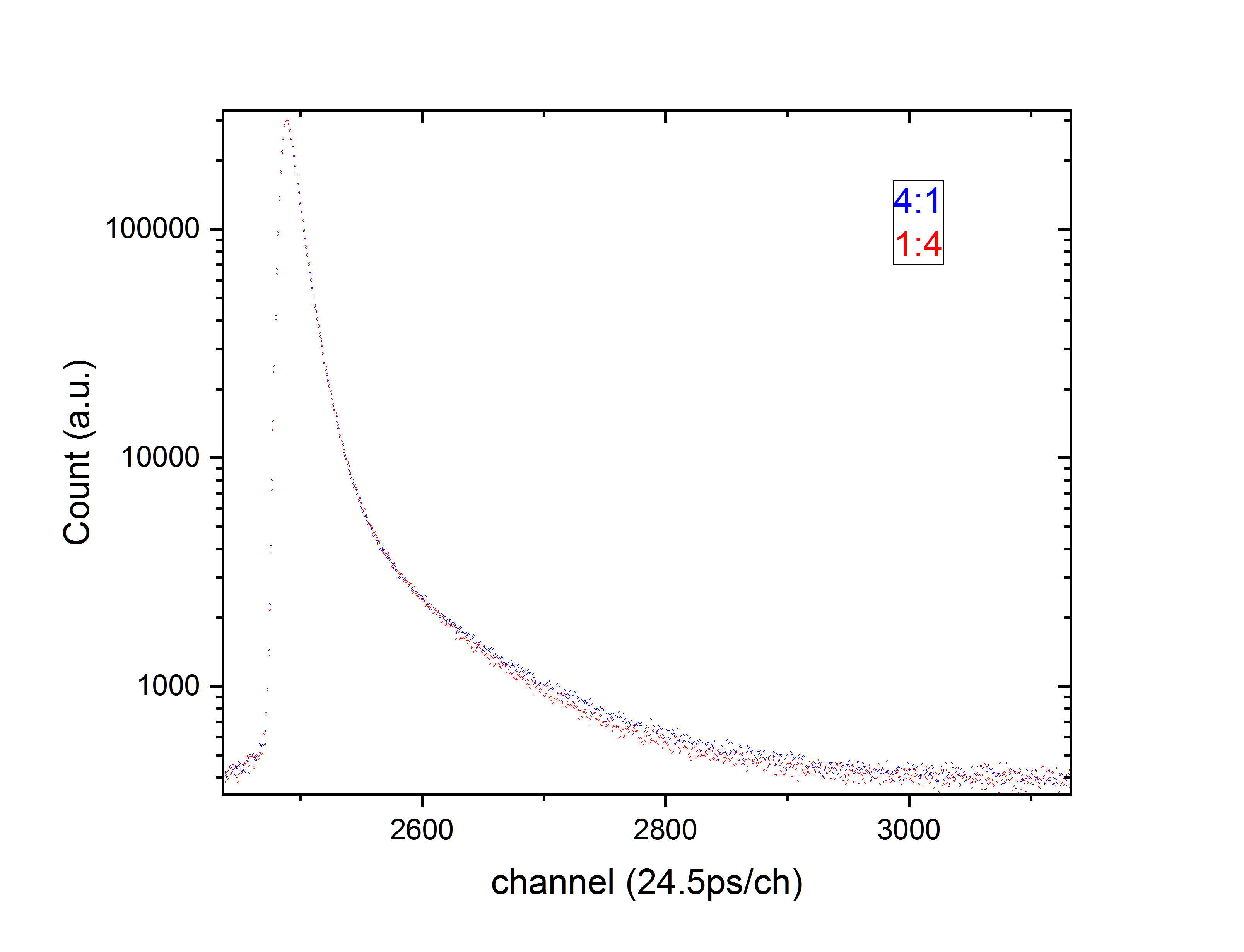


**Fig. S1** Representative PALS spectra for the mixture ratio 4:1 and 1:4.

1. **NIR – regions of double bonds (DB)**

Figure S2-S3 shows the absorption band of double bonds (6172.3 cm^-1^) selected at the fitting procedure for a serie of the cured samples with different ratio D_3_MA:UDMA after the baseline correction before photopolymerisation (liquid state) and after photopolymerisation (solid state). The peak areas of the double bond for different ratios before and after photopolymerization (Fig S4) were determined, on the basis of which the double bond concentration was determined (see Table 2).

**Fig. S2** Absorption of double bonds in the 6100 cm^-1^ regions of the samples with different ratios. before photopolymerisation (liquid state)

**Fig. S3** Absorption of double bonds in the 6100 cm^-1^ regions of the samples with different ratios after photopolymerisation (solid state).

**Fig. S4a, S4b** Peak area of the double bond of the liquid state (SI 4a) and the post-solidified state (SI 4b) for a sample with a 1:1 ratio.

The determination of the Area of the absorption band of double bonds we applied baseline correction in a selected region (6253.1 – 6110.63 cm^-1^) for each sample. Subsequently, we integrated spectrum to obtain the Area of double bonds.
